# Involvement of calpain in the neuropathogenesis of Alzheimer’s disease

**DOI:** 10.1002/med.21534

**Published:** 2018-09-10

**Authors:** Yacoubou Abdoul Razak Mahaman, Fang Huang, Henok Kessete Afewerky, Tanko Mahamane Salissou Maibouge, Bishwajit Ghose, Xiaochuan Wang

**Affiliations:** ^1^ Department of Pathophysiology, Key Laboratory of Education Ministry of China for Neurological Disorders School of Basic Medicine, Tongji Medical College, Huazhong University of Science and Technology Wuhan China; ^2^ Department of Social Medicine and Health Management, Tongji Medical College, Huazhong University of Science and Technology Wuhan China; ^3^ Division of Neurodegenerative Disorders Co‐innovation Center of Neuroregeneration, Nantong University Nantong China

**Keywords:** Alzheimer’s disease (AD), calpain, τ, amyloid beta, therapeutics

## Abstract

Alzheimer’s disease (AD) is the most common (60% to 80%) age‐related disease associated with dementia and is characterized by a deterioration of behavioral and cognitive capacities leading to death in few years after diagnosis, mainly due to complications from chronic illness. The characteristic hallmarks of the disease are extracellular senile plaques (SPs) and intracellular neurofibrillary tangles (NFTs) with neuropil threads, which are a direct result of amyloid precursor protein (APP) processing to Aβ, and τ hyperphosphorylation. However, many indirect underlying processes play a role in this event. One of these underlying mechanisms leading to these histological hallmarks is the uncontrolled hyperactivation of a family of cysteine proteases called calpains. Under normal physiological condition calpains participate in many processes of cells’ life and their activation is tightly controlled. However, with an increase in age, increased oxidative stress and other excitotoxicity assaults, this regulatory system becomes impaired and result in increased activation of these proteases involving them in the pathogenesis of various diseases including neurodegeneration like AD. Reviewed here is a pool of data on the implication of calpains in the pathogenesis of AD, the underlying molecular mechanism, and the potential of targeting these enzymes for AD therapeutics.

## 1 INTRODUCTION

Alzheimer’s disease (AD) is currently the most widely prevalent neurodegenerative disease affecting global health, mostly affecting aged individuals. The disease begins with a gradual loss in short‐term memory leading to a progressive impairment of cognition and behavior and finally a compromised ability of thinking, planning, judgment, and social skills.^1^ AD constitutes about 60 to 80% of overall dementia cases and is inevitably ending with the death of the affected individual in about 5 to 10 years after clinical diagnosis, mainly due to complications from chronic illness.^2^ The disease is marked histopathologically by extracellular deposit of amyloid beta (Aβ) surrounded by dystrophic neuritis forming senile plaques (SPs), and intracellular hyperphosphorylated τ made neurofibrillary tangles (NFTs).^3^, ^4^ The Aβ peptides are the result of proteolysis of the amyloid precursor protein (APP) by two proteases, the beta APP cleaving enzyme 1 (BACE1), also called β‐secretase, and the γ‐secretase,^5^, ^6^ and the hyperphosphorylated τ protein in the NFTs results from the dysregulation of kinase/phosphatase system, involving mainly glycogen synthase kinase 3β (GSK3β) and protein phosphatase 2A (PP2A) among other.^7^ Other key players in AD pathogenesis include oxidative stress seen as lipids; proteins and nucleic acid oxidations, neuroinflammation, calcium homeostasis disturbance. These inexorably lead to a decrease in PP2A and increase in GSK3β activities^8^, ^9^ which ultimately result in τ hyperphosphorylation and an increase Aβ production through increasing BACE1 activity via cyclin‐dependent kinase 5 (CDK5) activation by calpain.^10^ There is also an impaired autophagy/lysosomal clearance which results in the buildup and aggregation of harmful proteins including Aβ and τ.^11^, ^12^

Calpains are a family of cytoplasmic nonlysosomal cysteine proteases that are ubiquitously expressed in cells of human and other organisms. Calpains play a myriad of roles in calcium (Ca^2+^)‐regulated cellular functions.^13^, ^14^ Their enzymatic activity is under the regulation of Ca^2+^ and calpastatin (CAST) that activates and inhibits them, respectively.^14^, ^15^ The activation of calpains is controlled within smaller range, however, the stability of this regulating system tends to be impaired with increase in age resulting in an uncontrolled calpain activation,^16^, ^17^ implicating these enzymes in many disease conditions including neurodegenerative diseases like AD. Calpains are involved in the neuropathogenesis of AD where factors like oxidative stress and increased activation of *N*‐methyl‐d‐aspartate (NMDA) receptor play a role in intracellular calcium influx thus hyperactivating these enzymes. Upon activation, calpain mediates the cleavage of p35 to p25 (the most potent form of these CDK5 activators), GSK3β, dopamine‐regulated and cyclic adenosine monophosphate (cAMP)‐regulated phosphoprotein 32 kDa (DARPP‐32), promotes BACE1 synthesis, and APP truncation.^18^, ^19^, ^20^ In turn, the calpain‐mediated truncation of DARPP‐32 induced the dephosphorylation of a key protein involved in memory and learning and conversion of short‐term to long‐term memory^21^ known as cAMP‐response element‐binding protein (CREB).^18^ Calpain also cleaved lysosomal stabilizing protein like heat shock protein (Hsp) 70 and lysosome‐associated membrane protein 2a (LAMP2a).^22^, ^23^, ^24^ Together, these lead to Aβ plaques formation, τ phosphorylation, synaptic abnormalities, and memory and learning impairments observed in AD. In the present review, we first considered the types and functions of these proteases and how they are regulated in normal physiological conditions. We then reviewed data on the causes and consequences of the implication of calpains upregulation in AD pathogenesis. Lastly, we summarized available data on how calpain is targeted as potential AD therapeutics and the future direction.

## 2 TYPES AND BIOLOGICAL FUNCTION OF CALPAIN

**Figure 1:**
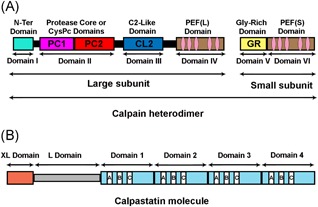
Structure of conventional calpains and calpastatin. A, Schematic representation of conventional calpains. Conventional calpains are heterodimer made up of a large catalytic subunit and a small regulatory subunit. The large subunit contains four domains which include: the anchor helix at the N‐terminus also called the N‐terminal domain or domain I, the catalytic CysPc domain made up of two protease core (PC1 and PC2) domains together constituting the domain II, the C2‐like domain and the penta‐EF‐hand (PEF[L]) domain, which contain the calcium‐binding sites play a regulatory role and constitute the domains III and IV, respectively. The small subunit contains a glycine‐rich (GR) domain and a PEF(S) domain similar to the large subunit, and together they make up the domains V and VI of the complete calpain heterodimer molecule. B, Schematic representation of the longest isoform of human calpastatin: the XL and L domains have no inhibitory activity, while the four inhibitory domains (1 to 4), each of them is capable of binding and inhibiting one calpain molecule. They are made up of A, B, and C subdomains or regions. While the subdomain B mediates calpain inhibitory effect of this molecule, the subdomains A and C interact with the PEF(L) and PEF(S) on the large and small subunits, respectively, and are required for the inhibitory effect of the B subdomain [Color figure can be viewed at wileyonlinelibrary.com]

Calpains are a family of widely distributed with highly conserved homology among species, Ca^2+^‐dependent proteases which were first discovered in 1964.^25^ It was given the name “calpain” to show its similarity with two other enzymes, the Ca^2+^‐regulated signaling protein, calmodulin “cal,” and the cysteine protease from papaya, papain “pain” (“cal” + “pain” = “calpain”). Although about 15 human genes (Table 1) of calpain family members are known to date,^13^, ^26^ only a few of them have attracted researchers’ attention. These mainly include the two isoforms, μ‐calpain and m‐calpain, reflecting their µM and mM Ca^2+^ requirement for activation, also known as calpain‐1 and calpain‐2, respectively, or conventional calpains, and their only and specific endogenous inhibitor CAST. Each of the μ‐calpain and m‐calpain form heterodimers composed of large and small subunits (Figure 1A). The large protease core subunit (80 kDa molecular weight) is encoded by the *CAPN1* gene for calpain‐1 and *CAPN2* gene for calpain‐2, with about 60% homology, while the shared small regulatory (28 kDa molecular weight) subunit is encoded by *CAPNS1* gene.^27^ These conventional calpains have a ubiquitous distribution, in contrast to some of other unconventional calpains which expression is limited to particular tissues. Characteristic examples are the expression of calpain‐3 in skeletal, calpain‐6 in embryonic muscles and placenta, calpains 8 and 9 in the gastrointestinal tract smooth muscles, calpain‐11 in the testes, calpain‐12 in the hair follicles, and calpain‐13 mostly in the lung and skin while others unconventional calpains, like calpain 5, 7, 9, and 10 have more ubiquitous distribution (Table 1).^13^, ^14^, ^28^

**Table 1:** Calpain family genes for human

Gene	Chromosome	Name	Distribution	Deficiency
*CAPN1*	11q13	CAPN1	Ubiquitous	Platelet dysfunction
*CAPN2*	1q41‐q42	CAPN2	Ubiquitous except erythrocytes	Embryonic lethality
*CAPN3*	15q15.1‐q21.1	CAPN3	Skeletal muscle	Muscular dystrophy
*CAPN5*	11q14	CAPN5	Ubiquitous	Vitreoretinopathy
*CAPN6*	Xq23	CAPN6	Embryonic muscles, placenta	Hypergenesis
*CAPN7*	3p24	CAPN7	Ubiquitous	⋯
*CAPN8*	1q41	CAPN8	Gastrointestinal tracts	Gastric ulcer
*CAPN9*	1q42.11‐q42.3	CAPN9	Gastrointestinal tracts	Gastric ulcer
*CAPN10*	2q37.3	CAPN10	Ubiquitous	Type 2 diabetes
*CAPN11*	6p12	CAPN11	Testis	⋯
*CAPN12*	19q13.2	CAPN12	Hair follicle	⋯
*CAPN13*	2p22‐p21	CAPN13	Ubiquitous	⋯
*CAPN14*	2p23.1‐p21	CAPN14	Ubiquitous	Eosinophilic Esophagitis
*CAPN15*	16p13.3	CAPN15	Ubiquitous	⋯
*CAPN16*	6q24.3	CAPN16	Ubiquitous	⋯
*CAPNS1*	19q13.1	CAPNS1	Ubiquitous	⋯
*CAPNS2*	16q12.2	CAPNS2	Ubiquitous	⋯
*CAST*	5q15	Calpastatin	Ubiquitous	⋯

The large subunit of the conventional calpains is divided into four domains (Figure 1A). Domain I (the *N*‐terminus) undergoes autolysis when Ca^2+^ binds to this molecule, domain II (the protease core or CysPc domain) forms the catalytic unit, domain III (the C2‐like domain) has a regulatory role, and domain IV (penta‐EF‐hand [PEF] domain) contains the Ca^2+^‐binding site.^29^, ^30^, ^31^ The shared small regulatory subunit has two domains a glycine‐rich (GR) domain and a calmodulin‐like PEF domain^32^, respectively, representing the (domain V) and (domain VI) of the calpain heterodimer (Figure 1A). CAST, the third most commonly studied member of the calpain system, is a heat‐stable unstructured protein which according to the type of the cell has a molecular weight ranging from ∼70 to ∼140 kDa with a ubiquitous widespread expression and is conserved (>70% identical) among mammalian species.^33^ The longest CAST molecule has six domains, four inhibitory homology units (CAST1‐4), and two *N*‐terminal domains, called XL domain and L domain (Figure 1B). Each of the four domains can specifically bind and effectively inhibit one calpain molecule, while the XL domain and L domain are devoid of any inhibitory potential.^34^, ^35^

Calpains have optimum activity at around neutral pH and are regulatory rather than digestive proteases. In normal physiological cell conditions, the concentration of Ca^2+^ is lower than 0.05 µM, a condition where calpains act as a biomodulator for Ca^2+^‐regulated processes, including among other signal transduction, cell proliferation, cell cycle progression, differentiation, apoptosis, learning, and memory and long‐term potentiation.^13^, ^14^, ^32^ It was previously thought that calpain cleavage sites have a Leu or a Val residue in the P2 position,^36^ but subsequent studies have shaded light on the fact that calpain‐mediated truncation of its substrates is not amino acid sequence‐specific but rather it is determined by the protein conformation.^37^, ^38^ Alterations in these proteases have been linked to some human diseases collectively known as calpainopathies, thus providing evidence of the physiological functions of calpains and the indispensable role they play. Supporting this are data that highlighted that deficiencies of calpain‐3 is associated with limb‐girdle muscular dystrophy 2A (LGMD‐2A),^39^, ^40^ calpain‐10 linked with diabetes mellites^41^, while recently calpain 5 mutation has been found to be associated with autoimmune uveitis and photoreceptor degeneration (vitreoretinopathy)^42^ and calpain‐14 has been associated with eosinophilic esophagitis.^43^

## 3 CALPAIN ACTIVATION

The enzymatic activity of calpains is under the regulatory control of Ca^2+^ that activates them and the endogenous protein CAST that inhibits them through a high‐affinity Ca^2+^‐dependent binding to the calpain.^14^, ^15^ When Ca^2+^ binds to the calpains it drives a conformational change that initiates their proteolytic properties. Calpain activity is also a measure of its autolyzed fragments.^44^ Besides induction of conformational changes, Ca^2+^ also induces calpains’ autoproteolytic cleavage that increases its protease activity. Most of the native µ‐calpain was found to be inactive until it undergoes autolysis and most of its activity is conferred to the 76 kDa fragment.^45^ In the presence of Ca^2+^, incubation of purified µ‐calpain with protein kinase A (PKA) or alkaline phosphatase (AP) increased its autolysis and therefore its activity^46^, ^47^ compared with control. A 6‐month chronic alcohol consumption in rats resulted in increased μ‐calpain autolytic activation in skeletal muscles.^48^ In another study in aging muscle, S‐nitrosylation of cysteine residues of μ‐calpain were observed and were suspected to be responsible for negatively regulating the autolytic activation of μ‐calpain as in the absence of nitrosylation agent the μ‐calpain is rapidly autolyzed due to the release of Ca^2+^ from sarcoplasmic reticulum.^49^, ^50^ Recently we have also reported an increase in calpain activity as seen by the increase in its autolyzed fragments in a hyperhomocysteinemia AD rat model.^51^ The presence of phosphatidylinositol was also reported to decrease the Ca^2+^ requirement for both µ‐calpain and m‐calpain autolysis in vitro.^52^ However, autolysis was also shown to lead to the loss of proteolytic activity of both µ‐ calpain and m‐calpain in high salt condition as they form aggregates into dimers and trimers.^53^ The half in vitro maximal Ca^2+^ requirement for activation of µ‐ calpain and m‐calpain, respectively, was reported to be around 3 to 50 µM and 0.4 to 0.8 mM which are significantly higher than the cytosolic concentration found in the living cells (<0.05 µM),^13^ suggesting that other mechanisms (interaction with phosphatidylinositol) are involved in the activation of these proteases. But still, the phospholipid/calpain molar ratio required for lowering the concentration of Ca^2+^ required for their activation is difficult to be achieved in the living cells.^54^, ^55^ Phosphorylation also plays a role as both conventional calpains were reported to be phosphorylated at multiple sites by kinases including PKA, protein kinase C, protein kinase G, calmodulin kinase II, and casein kinase I.^13^ Phosphorylation by extracellular signal‐regulated kinase (ERK) was believed to activate these proteases, specially calpain‐2 is activated via direct phosphorylation at its Ser50 by ERK.^56^ Supporting this were the findings that both epidermal growth factor (EGF) and brain‐derived neurotrophic factor (BDNF) could activate m‐calpain in hippocampal neurons dendrites by ERK‐mediated phosphorylation.^57^ Even though phosphorylation by PKA was demonstrated to increase µ‐calpain activity,^46^, ^47^ however, others reported a decrease calpains activity^58^ following PKA phosphorylation. It should also be noted that the dimerization of the two subunits is also important for the calpain activity as *CAPNS1* ^−/−^mice are embryonically lethal because both conventional calpains cannot dimerize with the small subunit indicating the crucial role of the small subunit in calpain stability.^59^

Calpains have more than 100 various substrates, however, their activation in normal physiological conditions is controlled within smaller range to prevent the devastating consequences of massive proteolysis of these numerous substrates. Unfortunately, these regulatory mechanisms decrease with increase in age leading to an upregulated calpains activity implicating them in multiple conditions ranging from muscular dystrophy, diabetes, traumatic brain injury (TBI) to neurodegenerative diseases including AD.

## 4 CALPAIN IN ALZHEIMER’S DISEASE

### 4.1 Overview

Elevated calpain activity as a result of disturbances in cellular Ca^2+^ homeostasis has been related to neuronal death in various neurotoxic conditions spanning from ischemia to AD.^60^ Aberrant uncontrolled calpain‐mediated proteolysis has been associated with various neurodegenerative diseases including AD.^13^, ^14^, ^61^ τ Hyperphosphorylation and Aβ accumulation are the main markers of AD. These were found to be promoted in neuronal cells through Ca^2+^ dysregulation.^62^ These Ca^2+^‐regulated proteases “calpains” were abnormally activated and abnormally high in AD patients’ brains.^63^ Other reports have shown evidence of calpain involvement in the AD pathogenesis whereby the amount of m‐calpain in the cytosolic fraction of brain from AD patients is increased,^13^ the ratio of autolyzed to unautolyzed µ‐calpain threefold higher, and amount of CAST lowered in AD prefrontal cortex than in normal brains.^61^, ^64^ This decrease in CAST, which results from calpain and caspase proteolysis^64^ led to a subsequent decrease in the CAST/calpain ratio therefore increasing calpain activation.^44^, ^65^ Using APP overexpressing neurons, a study demonstrated an increased activation of caspase‐3 and calpain as shown by the calpain‐cleaved high levels of α‐fodrin and autolyzed µ‐calpain fragments while these two proteases were not activated in APP mutant neurons. Furthermore, E64d a membrane permeable calpain inhibitor almost completely inhibited the neuronal caspase‐3 activation and neuronal death induced by APP, an indication that high amount of wild‐type APP might induce neuronal apoptosis through calpain‐mediated caspase‐3 activation in postmitotic neurons.^66^

Upregulation of calpain activation in the brain of AD activates CDK5 through cleavage of p35 to p25^67^ and its homolog p39 to p29^60^ by excitotoxicity‐activated calpain and CDK5 activates BACE1 promoter by binding to its target STAT3, therefore increasing BACE1 expression, Aβ40 and Aβ42 production in transgenic mice.^68^ Thus, calpain overactivation leads to the two AD hallmarks, Aβ aggregates leading to SP and τ hyperphosphorylation leading to NFTs. The high amount of calpain‐cleaved spectrin detected in AD patients’ cerebrospinal fluid (CSF) is also suggestive of the hyperactivated calpains in the brains of these patients.^63^ Abnormal calpain‐2 but not calpain‐1 activation was shown to be increased in NFTs and induces degradation of nicotinic acetylcholine receptor α4 thus impairing cholinergic system in AD.^69^, ^70^

With age the central nervous system (CNS) becomes more and more susceptible to oxidative stress with widespread consequences on proteins, lipids, and nucleic acids.^71^, ^72^ As glutamate is the main excitatory neurotransmitter in the CNS, oxidative stress‐induced glutamatergic excitotoxicity lead to uncontrolled hyperactivation of NMDA receptors which increased neurons permeability to Ca^2+^ and therefore injuring them. Interestingly NMDA receptor antagonists’ treatment reduced the glutamatergic excitotoxicity associated damage.^73^ The resulting Ca^2+^ influx in the neurons induced an increase calpain activation which through increase production of Aβ^74^, ^75^ also increases the oxidative stress thus making a circle damaging more and more neurons resulting in observed neurodegeneration in the late stage of AD.

Inflammation seen as reactive astrocytes is also a marker of AD. In line with this a study found αΙΙ spectrin breakdown product (BDP) positive astrocytes in progressive activation stages, and this spectrin BDP persists till fully reactive astrocytes. This is suggestive of the likelihood of calpains and their spectrin proteolytic activity involvement in the physiological and morphologic transformation of astrocytes from resting to reactive fibrous astrocytes.^76^ In support of this overexpressing CAST in a mouse model of PD resulted in reduced calpain activity and decreased gliosis^77^ and calpain inhibition by A‐705053 decreased the astrocytic and microglial reactivity associated with AD‐like pathology in aged 3xTg‐AD mice.^78^ Furthermore, Aβ deposit was found to be in association with neuronal and astrocytic Ca^2+^ dysregulation.^79^, ^80^ Treatment of cortical neurons with Aβ oligomers led to Ca^2+^ influx resulting in calpain activation.^67^ All these highlighted the role that calpain plays in the neuroinflammation associated with neurodegenerative diseases.

### 4.2 Calpain in Aβ pathology

Aβ aggregates is one of the hallmarks of AD and Aβ oligomers aggregation process which was reported to adversely affect synaptic structure and plasticity^81^, ^82^ highlighting the observed cognitive impairments in AD. Aβ aggregation was found to be in association with neuronal and astrocytic Ca^2+^ disturbances.^79^, ^80^ Many studies have provided evidence of the increased level and/or activity of BACE1 upon a variety of assaults like oxidative stress,^83^ hypoxia,^84^ ischemia^85^, and TBI.^86^ Likewise, the level and activity of BACE1 were reported to be upregulated in AD brain,^87^, ^88^ and this might initiate or at least worsen AD progression. On another hand the level of BACE1 and the corresponding β‐carboxy terminal fragment (β‐CTF) of APP were decreased in CAST overexpressing APP/PS1 mice, meaning that this could be a mechanism via which calpain activation might promote APP processing through BACE1 synthesis.^20^ In support of this, is the observation that BACE1 protein levels were increased by m‐calpain overexpression in HEK293 cells expressing APP^20^, and also calpain activation affected APP processing as shown by increased β‐CTF production.^89^ However, various calpain inhibitors led to opposite effects.^20^ This indicates that downregulation of calpain suppressed the levels of BACE1, which ultimately decreased APP proteolysis and Aβ generation. BACE1 upregulation could be mediated by CDK5, which is calpain regulated through truncation of p35 to p25.^74^ CDK5 activates BACE1 promoter by binding to its target STAT3, therefore increasing BACE1 level and activity, Aβ40 and Aβ42 production in transgenic mice.^68^ Another experiment also reported that upregulation of calpain and p25/CDK5 resulted in phosphorylation of STAT3 (pSTAT3) which led to increase in both BACE1 and presenilin‐1 (component of γ‐secretase) transcription both of which are implicated in Aβ production.^90^ Of particular interest is the observation that p25, a product resulting from p35 cleavage by calpain, induces the production and accumulation of Aβ in vivo,^75^ presumably by increasing BACE1 transcription.^68^ The phosphorylation of APP at Thr668 is believed to play a critical role in its proteolytic cleavage into soluble Aβ producing fragment. The main kinase to phosphorylate APP at this site was shown to be CDK5,^91^ suggesting that the calpain‐mediated activation of CDK5 also leads to the amyloidogenic APP processing both via BACE1 expression and APP Thr668 phosphorylation. Thus, calpain activation triggered by various stressors including oxidative stress, excitotoxicity, and Aβ among other might increase the level of BACE1, which in turn promotes processing of APP and more Aβ production. In turn the upregulated Aβ levels lead to increased Ca^2+^ influx and therefore increasing calpain activation.^63^

It has been reported that extracellular Ca^2+^ influx can be triggered by Aβ through stimulation of membrane ion channels or receptors, such as ionotropic glutamate receptors.^92^ Aβ could also impair the distribution of intracellular Ca^2+^ by increasing membrane permeability to Ca^2+^ via oxidative stress.^93^, ^94^ It was also found that in familial AD, some presenilin mutations, linked to increased Aβ levels, impaired Ca^2+^ homeostasis by deregulating voltage‐gated internal Ca^2+^ channels ryanodine receptor,^95^, ^96^ inositol 1,4,5‐triphosphate (IP3) channel,^97^ or sarcoendoplasmic reticulum Ca^2+^ ATPase (SERCA).^96^, ^98^ Increased Ca^2+^ in hippocampal neurons has been observed and thought to originate from endoplasmic reticulum leading to calpain hyperactivation^99^ and BAPTA a Ca^2+^ chelators was shown to inhibit calpain activation.^100^ Aβ was reported to enhance calpain activation by inducing extracellular Ca^2+^ influx^99^ and NMDA receptors has been found to play a role as shown by studies where M7K801 a specific NMDA receptor inhibitor^101^ and memantine an NMDA receptor antagonist^102^ were reported to attenuate the Aβ‐induced Ca^2+^ influx thereby blocking calpain activation in hippocampal cultured neurons.^99^ Cholesterol a risk factor for AD^103^ might also play a role in Aβ‐mediated calcium influx through influencing NMDA receptors components and their membrane microdomain localization at synaptic sites.^104^ Aβ was also found to activate NR2B‐containing NMDA receptors.^105^

Another way Aβ affect Ca^2+^ is through the βAPP intracellular domain (AICD) which was shown to be involved in Ca^2+^ signaling^106^ or homeostasis in different cell culture models.^107^ Advance glycation end products (AGEs) influence Aβ generation through induction of βAPP expression via oxidative stress in SHSY5Y cells^108^ while injection of receptors for AGEs (RAGE) in AD transgenic mice model increases Aβ accumulation and SP formation.^109^ The same study revealed an increased BACE1 expression and activity by RAGE which was mediated by AGEs or Aβ stimulation of RAGE thus increasing cytosolic Ca^2+^ concentration and finally activating the transcription factor NFAT1 which leads to increased transcription of BACE1 in SHSY5Y cells.^109^ The increased cytosolic Ca^2+^ might also activate calpain.

Together, the data reviewed here suggest that in AD calpain and Aβ form a closed circle whereby Aβ leads to the activation of calpain, which in turn leads to a feedforward process to produce more Aβ and therefore more calpain activation.

### 4.3 Calpain in τ pathology

Calpain is also involved in AD by favoring τ pathology including indirect‐mediated phosphorylation and direct‐cleavage favoring aggregation. Potential candidate τ protein kinases include CDK5^110^ and GSK3.^111^ Both CDK5 and GSK3β were shown to be activated by calpain. Studies have shown that, τ phosphorylation is achieved through increased activation of both CDK5 and GSK3β, the main kinases of τ, and decreasing PP2A activity, the main phosphatase of τ. Several kinases induce τ phosphorylation at over 38 Ser/Thr residues, these include ERK, CDK5,^112^ GSK3β,^113^ calcium/calmodulin‐dependent protein kinase II (CaMKII),^114^ PKA,^115^ the dual‐specificity tyrosine phosphorylation‐regulated kinase‐1A (Dyrk1A),^116^ mitogen‐activated protein (MAP) kinase^117^, and stress‐activated protein kinases (SAPKs)^118^ among others.

The most elucidated pathway of calpains involvement in AD is the calpain‐mediated proteolysis of p35 to a 25 kDa form p25. Calpain was reported to cleave p35, an activator of CDK5, and generates p25^20^, ^67^, ^74^, ^78^ which has longer half‐life than p35, and therefore is a more potent CDK5 activator, leading to a sustained activation of CDK5 and causes phosphorylation of many of its substrates. Since τ is a substrate of CDK5, it is not surprising that the p25‐mediated activation of CDK5 led to τ hyperphosphorylation and the resulting NFTs formation. Furthermore, this p25 was found to accumulate in brains of AD patients,^67^ suggesting that calpain‐cleaved p25 is at least in part responsible for the hyperphosphorylation of τ in the intracellular NFTs observed in AD brains.^119^ Moreover, the production of p25 resulted in an increased Aβ level in forebrain before any sign of neuropathology in APP transgenic mice^75^ probably through upregulation of BACE1 expression.^68^ Abnormal τ phosphorylation was observed in the APP/PS1^120^ and this was prevented by overexpressing CAST^20^ also suggesting calpain intervention.

GSK3β the other main τ kinase was also reported to be activated by calpain.^19^, ^121^, ^122^ Incubation of mouse brain tissue homogenates with CaCl_2_ was found to trigger the cleavage of GSK3β in two fragments of 40 and 30 kDa in a time‐dependent way, while these cleaved fragments disappeared in the absence of Ca^2+^.^19^ Compared with control, this Ca^2+^‐mediated proteolysis increased the GSK3β kinase activity. Furthermore, this cleavage and activation of GSK3β were inhibited by calpeptin, a calpain inhibitor, not other protease inhibitors, suggesting that this breakdown and increased activity may be due to Ca^2+^‐dependent calpain activation.^19^ It should be noted also that phosphorylation of GSK3β at Ser9 abolished the calpain‐mediated cleavage at both C and N‐termini while phosphorylation at Ser‐389 only inhibit N‐terminal cleavage.^122^

CaMKII can undergo autophosphorylation (activation) in the presence of Ca^2+^,^123^ was shown to be a τ kinase and has a role in memory and neuroplasticity.^114^ However, its redistribution or prolonged activation could implicate it in AD. In support, a correlation with cognitive dysfunction in MCI and AD subjects was found with the reduction of T286‐autophosphorylation in apical dendrites of granule cells of the DG.^124^ Resveratrol was shown to decrease τ hyperphosphorylation via suppression of GSK3β and CaMKII activities in N2A cells exposed to formaldehyde.^125^ Strong and sustained stress‐like acute severe CO poisoning could lead to both calpain and CaMKII expression and activation.^126^ Calpain might also induce Ca^2+^ as revealed by studies where increased expression of µ‐calpain resulted in mitochondrial damage^127^ with consequent production of reactive oxygen species (ROS) which in turn resulted in a significant Ca^2+^ increase^128^ and thus activation of CaMKII. Via membrane skeleton protein degradation calpain was also shown to be upstream activator of CaMKII resulting in ischemia/reperfusion (IR) injury.^129^ Interestingly CaMKs family are shown to be substrates of calpain. More specially calpain‐mediated CaMKIV activation is characterized in the apoptosis of SHSY5Y cells^130^ and cerebellar granule cells,^131^ while m‐calpain was shown to activate CaMKII‐gamma which in turn led to cAMP/PKA‐mediated ERK1/2 phosphorylation resulting in caspase‐3–mediated apoptosis in head kidney macrophages.^132^ It should also be noted that CaMKII plays an antiapoptotic.

PKA is also reported to be a τ kinase as revealed by the study where cAMP‐dependent PKA phosphorylation of τ at serine 214 was observed in monkey^133^ and SHSY5Y cells.^134^ PKA activator forskolin was shown to induce τ phosphorylation at Thr231.^115^ In addition, an in vitro study showed that in the presence of Ca^2+^ incubation of μ‐calpain with PKA resulted in more autolysis than control, but lower than the AP group. Furthermore, PKA was found to catalyze μ‐calpain phosphorylation at serine 255, 256, and 476, and upregulated μ‐calpain activity.^46^, ^47^ Together this could lead to the downstream of calpain including Aβ and τ pathologies. However, another study showed that the degradation of calpain substrate proteins was higher in AP‐treated sample and less in PKA‐treated ones.^135^

Both APP and τ were reported to be substrates of Dyrk1A, therefore Dyrk1A is linked to the two main hallmarks of AD. Dyrk1 inhibition rescued the observed cognitive deficits in 3xTg‐AD mice with a concomitant reduction in Aβ and τ pathology^136^ via decreasing phosphorylation of both APP and insoluble τ, thereby increasing APP metabolism and decreased Aβ levels.^137^ DYRK1A phosphorylates τ on 11 different Ser/Thr residues many of which are found in NFTs of AD,^116^ and its overexpression led to increased 3R‐τ and decreased 4R‐τ.^138^ This is mediated through influence on the alternative slicing of τ exon 10.^139^, ^140^ Inhibition of Dyrk1A by Leucettine L41^141^ or EHT 5372^142^ mitigated both τ and Aβ pathologies. Interestingly Dyrk1A was reported to be truncated at the C‐terminus and this was associated with increased µ‐calpain activity in AD brain. Moreover, in vitro studies revealed that µ‐calpain proteolyzed Dyrk1A at both C and N termini and enhanced its τ kinase activity, with C‐terminus truncation producing Dyrk1A with a stronger activity than its full‐length protein in promoting τ phosphorylation and exon 10 exclusion.^143^

Creatine kinase (CK) is found in many tissues including brain, cardiac, and skeletal muscles. The creatine/phosphocreatine system which is under the regulation of CK, plays an important role in energy metabolism therefore very important for neuron. It is well known that energy metabolism and the CK function are affected in AD brain and in cells exposed to the Aβ peptide.^144^ Creatine was also focally detected in hippocampi from APP transgenic mice and postmortem AD patients brain and was suggested to be a marker of the disease,^145^ probably due to a decreased CK activity. Supporting this, other studies also found a decrease in this enzyme and proposed that this could due to oxidative damage as CK is sensitive to oxidative stress.^146^, ^147^ Moreover, CK was also found to be a calpain substrate.^148^ Accordingly, another study reported a decrease in CK activity with increased calpain activity^149^ suggesting the degradation of this enzyme by calpain. Furthermore, calpain inhibition was shown to prevent cell death in IR, via decreasing DNA strand breaks and CK release, suggesting calpain is an upstream of CK.^150^, ^151^

Calpains also activates ERK/MAP kinases^152^ and other kinases that are also involved in τ phosphorylation. Both calpain and ERK1,2 were found to be activated along with concomitant neurofilament phosphorylation and interestingly the inhibition of calpain by calpeptin abolished both neurofilament phosphorylation and ERK1,2 activation, while inhibiting ERK1,2 by a specific inhibitor Meck1,2 had no effect on calpain activation suggesting calpain to be an upstream activator of the ERK/MAP pathway.^152^

To induce τ phosphorylation, calpain could also directly or indirectly affect phosphatases. PP2A account for most of the dephosphorylation activity of τ, however protein phosphatase 1 (PP1) also play a part. Therefore, even though it was shown to be neuroprotective, the degradation of PP1 mediated by µ‐calpain^153^ might increase τ phosphorylation as seen in AD. Calpain was also shown to mediate the cleavage of the PP2A regulatory subunit α4 leading to the proteasomal degradation of PP2Ac resulting in τ hyperphosphorylation.^154^ GSK3β one of the main kinase of τ is known to be truncated by calpain leading to its increased activity. The underlying mechanism is reported to be its translocation to the nucleus and its enhanced interaction with PP2A upon truncation, promoting its Ser9 dephosphorylation and thus increasing its activity^155^ highlighting an indirect intervention of calpain on PP2A‐mediated activation of this kinase and involvement in AD. Also, extra‐synaptic *N*‐methyl‐d‐aspartate receptor (NMDAR) activation led to neuronal death as a result of degradation of striatal‐enriched protein tyrosine phosphatase (STEP) mediated by calpain.^153^ Tyrosine phosphatase (PTPN13) is reported to dephosphorylates and inhibits c‐Abl (τ kinase) but also to be a substrate of m‐calpain which inactivates its phosphatase activity.^156^ Calpain was also reported to induce the degradation of inositol polyphosphate‐4‐phosphatase (INPP4),^157^ a negative regulator of the phosphoinositide 3‐kinase (PI3K) signaling pathway.^158^ PI3K is known to phosphorylate GSK3β at Ser9 inhibiting its kinase activity.^159^ Thus, by degrading INPP4 and PTPN13, calpain might respectively increase the kinase activities of GSK3β and c‐Abl, therefore increasing τ phosphorylation and accumulation.

Besides τ phosphorylation, another way calpain activation play a role in τ‐mediated neurodegeneration is through inducing τ cleavage.^160^, ^161^, ^162^, ^163^, ^164^ But this cleavage might be at earlier stage of the disease since hyperphosphorylation makes τ, which normally is rapidly cleaved by the calpains,^13^, ^165^ highly resistant to calpain truncation,^166^, ^167^ so the NFTs in AD are resistant to calpain^168^ suggesting phosphorylation might regulate τ susceptibility to calpain. Hence, because neurofilament proteins are good substrates of calpains, it may play an important role in the AD observed necrotic neuronal death. Therefore, small fragments of τ, which were believed to be generated by calpain‐1 proteolysis, have been observed in Aβ‐treated hippocampal neurons and these fragments of around 17 kDa (τ 45 to 230) are neurotoxic.^162^, ^163^ Another study also reported the presence of these types of fragments in the neocortex of AD brains as well as in brain areas affected by other tauopathies and their levels correlated with increased calpain activity.^167^ Furthermore, these 17 kDa τ fragments induced neuronal cell death even in the absence of Aβ oligomers following their expression in neuronal and nonneuronal cell types or in an in vivo drosophila model system.^162^, ^163^, ^169^ Fragmented τ has also been found in mitochondria present in synaptosomal fraction from AD brain and the level of these fragments were found to be reduced in culture neurons treated with calpain inhibitors.^160^ These types of fragments were reported to induce mitochondrial dysfunction.^164^ By the way it could be also noted that calpain‐2 activation generates smaller τ fragments with nonobservable neurotoxic effects in CNS neurons.^161^

Collectively, these data strongly provided evidence of the deleterious role of calpain in τ hyperphosphorylation and generation of neurotoxic τ fragments in neurodegeneration.

### 4.4 Calpain in autophagy/lysosomal impairment

Impaired autophagy/lysosomal pathway has been implicated in AD resulting in the buildup of vacuoles containing harmful proteins^11^, ^12^ seen as granulovascular degeneration. Oxidative stress is clearly connected to autophagic pathway dysregulation as a result of ROS‐mediated carbonylation of proteins and eventually lead to neuronal dysfunction, degeneration, and cell death that is seen in many neuropathological conditions including AD. Hsp70.1 is one of the major component of the human Hsp70 proteins family which are chaperones that help the cell get rid of harmful aggregated denatured proteins in various environmental insults such as heat, oxidative stresses, and ischemia.^170^, ^171^ AD brains were reported to show progressive ischemic and oxidative assaults that lead to Ca^2+^ homeostasis dysregulation and calpain activation. Another way Hsp70s confer their cellular protection is via inhibition of rupture/permeabilization of lysosomal membrane,^172^, ^173^ thus maintaining lysosomal membrane integrity and are essential for both endosomal and lysosomal autophagy. Elevated levels of Hsp70 carbonylation was reported in the brain of mild cognitive impairment which is an early phase of AD^174^ and in hippocampal CA1 in IR brain.^175^ Interestingly Hsp70.1 is found to be an in vivo^22^ and in vitro^23^ substrate of calpain. Hsp70.1 has been demonstrated to be more prone to the calpain cleavage following carbonylation by oxidative stressors, such as HNE or H_2_O_2_.^176^ Therefore calpain‐induced Hsp70.1 truncation together with ROS‐mediated Hsp70.1 carbonylation, lead not only to impaired autophagy and buildup of harmful proteins but also to lysosomal rupture/permeabilization leading to release of lysosomal enzymes and cell death in both IR and AD brains.^23^, ^176^, ^177^ Moreover, LAMP2a a molecular marker for lysosome stability and an essential component of chaperone‐mediated autophagy,^178^ was also reported to be cleaved by calpain leading to lysosomal rupture and cell death.^24^ Also, downregulation of LAMP2a via RNAi, led to impaired lysosomal degradation of long‐lived proteins and chaperone‐mediated autophagy substrates in neuronal systems.^179^ Hsp70.1 was also shown to attenuate τ toxicity via maintaining τ in its soluble nonaggregated nature and by facilitating its degradation.^180^, ^181^, ^182^ Thus, impaired Hsp70.1 function as a result of carbonylation and/or cleavage, as a result of calpain activation, could lead not only to lysosomal destabilization but also τ aggregation and NFTs formation.^182^, ^183^

Together these suggested that cleavage of Hsp70.1 and LAMP2a is another way that calpain contributes to neurodegenerative changes in AD and related conditions.

### 4.5 Calpain in synaptic dysfunction

Among calpain substrates are included both presynaptic and postsynaptic proteins, so it is not surprising that calpain is implicated in synaptic dysregulation. Accumulating evidence have been provided by many studies as per the abnormal activation of calpains not only favors Aβ accumulation and τ hyperphosphorylation in neurons but also is implicated in the development of synaptic pathology in neurodegenerative diseases including AD.^77^, ^78^, ^184^ Overactivation of calpain in the brain of transgenic mice, induced a myriad of AD pathological markers including βAPP processing, amyloid deposits, τ phosphorylation, activation of astrocytes, synapse loss, and cognitive impairment.^20^, ^63^, ^77^ These synaptic dysfunctions seen as synaptic loss seems to significantly correlate with the functional and cognitive deficits observed in different stages of AD.^185^ Supporting this is a report from a study, where the calpain involvement in synaptic loss is highlighted by its cleavage of proteins in the presynaptic termini and/or the postsynaptic elements, thus leading to functional changes in affected brain areas.^186^ Using cell culture and animal models of AD Aβ was recently shown to induce a significantly reduced level of dynamin‐1, a neuron‐specific GTPase highly enriched in presynaptic terminals which function is to detach synaptic vesicles from the membrane so that they can reenter the synaptic vesicle pool and be refilled for future release.^187^ This decrease in dynamin protein lead to the depletion of synaptic vesicles and the accumulations of empty pouches at presynaptic termini, thus leading to synaptic dysfunction. This Aβ‐mediated dynamin‐1 reduction was believed to be in part due to calpain‐mediated proteolysis.^99^, ^188^ At the postsynaptic element, calpain hyperactivation resulted in the degradation of several proteins changing the ultrastructure of the postsynaptic density (PSD). Following this event are the accelerated cleavage of PSD95 and the NMDA receptor subunits NR1 and NR2A and 2B.^189^, ^190^ Calpain was shown to also exacerbate NMDA neurotoxicity by cleaving mGluR1.^191^, ^192^ Additionally, calpain activation induces the cleavage of PKA leading to a decrease in both its regulatory and the catalytic subunits thus decreasing the activity of CREB, an important memory‐related protein, aggravating synaptic dysfunction^193^ as seen by the resulting memory impairment. In a mouse model of PD overexpressing CAST the observed decreased calpain activity was found to be accompanied by a concomitant reduction of the cleaved synaptic protein α‐synuclein ameliorating the observed neurodegeneration.^77^ In support, using calpain inhibitor A‐705053 treatment in 3xTg‐AD mice model significantly increased the levels of synapsin‐1 and PSD95 to nontransgenic animal level thereby ameliorating the synaptic loss observed in these AD mice model.^78^

All of these revealed the undeniable role that calpain played in synaptic pathologies.

### 4.6 Calpain in learning and memory

Many studies have provided data concerning the calpain‐mediated dysregulation of learning and memory‐related molecules in AD including ERK, CREB, and DARPP‐32 among others. CREB, a key player in learning and memory and synaptic plasticity, and a core component involved in converting short‐term to long‐term memory,^21^ has been found to be dysregulated in various neurological disorders, including AD, Huntington’s disease, Parkinson’s disease, ischemia, and addiction.^194^, ^195^ In line with this ERK and CREB phosphorylation (activation) were reported to be decreased in transgenic AD mouse model,^20^, ^184^ while overexpression of the endogenous calpain inhibitor CAST prevented this decrease with concomitant improvement in learning and memory ability in these animals.^20^ Also, selectively inhibiting calpain‐2 by C2‐I resulted in an improved state of learning and memory through increasing timely activation of ERK.^196^ DARPP‐32 is a key player involved in the regulation of the PKA‐CREB signaling pathway.^197^ An increase in the cAMP level leads to the activation of PKA and phosphorylation of DARPP‐32 at Thr34, which inhibits PP1 a key phosphatase of CREB, therefore increasing CREB phosphorylation.^198^ Interestingly, in AD brains DARPP‐32 protein level was found to be about 20% lower compared with control brains, however two fragmented forms, about 28 and 4 kDa, of DARPP‐32 were found in high amount in AD brains.^18^ Cleavage of DARPP‐32 protein was also reported in both OA or Aβ peptides treated SHSY5Y cells and primary neurons along with a simultaneous decrease in CREB phosphorylation ultimately resulting in synaptic abnormalities.^18^ Consistently, calpain inhibition reversed the DARPP‐32 truncation in the OA‐treated neurons while recombinant DARPP‐32 was cleaved by calpain in an in vitro assay.^18^ In support of this, restoring CREB phosphorylation through calpain inhibition has been reported to ameliorate synaptic transmission and memory in an AD mouse model.^184^ This is in accordance with the findings that calpain affects CREB kinases such as PKA and CaMKII.

These data together suggested that in AD, calpain‐mediated DARPP‐32 truncation and activation of PP1 might be involved in the impairment of the PKA‐CREB signaling pathway and therefore impairing learning and memory ability in the affected subject. It is worth noting that, in normal physiological conditions, calpain also play an important role in synaptic plasticity and thus a positive regulator of learning and memory.^199^, ^200^ Recently it was reported that calpain‐1 and calpain‐2 are implicated in learning and memory via two opposing effects where calpain‐1 was found to positively affect LTP and learning and memory while calpain‐2 restricted the extent of learning and memory.^196^, ^201^ Thus, calpains are essential for life but their uncontrolled hyperactivation is destructive and implicate them in the etiopathology of many diseases conditions including AD.

## 5 CALPAIN INHIBITORS AS AD THERAPY

**Figure 2:**
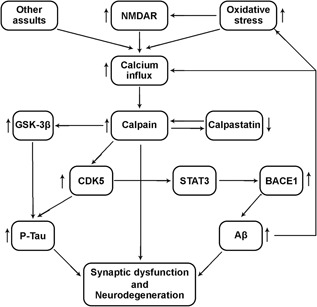
Summary of calpain involvement in AD pathology. Intracellular calcium influx resulting from increase oxidative stress, increased NMDAR activation and other assaults together lead to increased and uncontrolled calpain activation. This calpain hyperactivation could also result from impaired regulatory mechanisms like decreased calpastatin level which could also be a result of calpain proteolysis. The resulting calpain upregulation then lead to truncation and increased GSK3β activation, cleavage of p35 to p25 with resulting prolonged CDK5 activation. On one hand these two (GSK3β and CDK5) lead to τ hyperphosphorylation, while on the other hand, the prolonged CDK5 activation lead to increase BACE1 expression and activation via STAT3 with consequent increased Aβ production. The τ hyperphosphorylation and Aβ deposit together lead to synaptic dysfunction and neurodegeneration. The Aβ peptides could also exert a positive feedback leading to increased oxidative stress and/or increased calcium influx and more calpain activation and the cycle continues. AD, Alzheimer’s disease; Aβ, amyloid beta; BACE1, beta APP cleaving enzyme 1; GSK3β, glycogen synthase kinase 3β; NMDAR, *N*‐methyl‐d‐aspartate receptor

The undeniable role that calpains play in AD progression and pathogenesis made it a potential target for therapeutics, and thus has stimulated the development of many calpain inhibitors for AD treatment. Various insults to the CNS activate calpain, leading to Aβ production, τ hyperphosphorylation, synaptic dysfunction, and neuronal degeneration.^13^ Upregulated calpain activity due to Ca^2+^ influx or decrease of CAST causes the proteolysis of many functional proteins in the brain.^84^, ^202^, ^203^ Together, this implies that calpain play a multistage role in AD progression from the earlier APP processing via BACE1 activation to the later CDK5 and GSK3β activation, the resulting τ hyperphosphorylation, autophagy impairments, synaptic and neuronal dysfunction, and the finally observed neurodegeneration (Figure 2). Thus, calpain inhibition could be a potential option in preventing neuronal degeneration induced by multiple neurotoxic factors.

A study by Trinchese et al^184^ showed that synthetic calpain inhibitors improved memory and synaptic transmission without significant effects on Aβ deposit in APP/PS1 mice. Considering that synthetic calpain inhibitors are not highly specific, they might affect other proteases and therefore may influence neurodegeneration processes. Thus, inhibiting calpain by overexpressing its endogenous inhibitor CAST would be a better way to study isolated calpain effect. Interestingly, CAST overexpression in APP/PS1 (APP/PS1/CAST),^20^ APP23 (APP23/CAST),^204^ APP (APP/CAST),^63^ and JNPL3 (CAST/JNPL3)^205^ mice led to a significant decrease in Aβ level and/or τ phosphorylation and this effect appeared to be mediated through decreasing BACE1 and β‐CTF levels. ThisCAST‐mediated inhibition of calpain in the nervous system is more responsive in pathological conditions.^204^ Similar effects with CAST overexpression have also been demonstrated with synthetic inhibitors as they also suppressed calpain and reduced BACE1 levels, which attenuated APP processing and therefore Aβ generation.^20^ However, stimulation and activation of calpain by various stressors^20^ or CAST knock‐out^63^ caused the opposite effects. Additionally, the CAST overexpression also prevented astrocyte activation, microgliosis, and synapse loss.^20^, ^63^ APP/PS1 mice showed an increase in AT8‐positive phosphorylated τ protein in their brain in comparison with wild‐type control mice, while overexpressing CAST abolished it^20^ and synthetic calpain inhibitors were reported to improve synaptic transmission and memory in AD mouse model.^184^ Synthetic calpain inhibitors also showed neuroprotective effect in neuronal injuries like ischemia and excitotoxicity‐induced neuronal death.^64^, ^206^ To date many calpain inhibitors have been synthesized owing to the role played by this enzyme in AD pathogenesis. These inhibitors include compounds like SNJ‐1945, a compound with good metabolic stability and permeability,^207^ and has been shown to decrease in vitro and in vivo death of retinal cell.^208^, ^209^ MDL28170, another calpain inhibitor, has been reported to have some protective effects against excitotoxicity.^191^, ^210^ E64, BDA‐410, and leupeptin calpain inhibitors have also showed protective effects in AD cell culture models.^78^, ^162^, ^211^, ^212^ Other inhibitors include SJA6017, ALLN,^207^ and a recently produced A‐705053.^213^ This novel calpain inhibitor A‐705053, which can easily be taken orally, has improved pharmacokinetics and has provided interesting results as it was shown to prevent calpain‐mediated truncation of dynamin‐1 and τ in primary hippocampal neurons culture.^188^ It was also effective when used as preventive, simultaneous, or curative treatment against Aβ‐triggered neurodegenerative process^188^ and prevented neurodegeneration of the nucleus basalis magnocellularis induced by Aβ oligomer.^214^ A‐705053 was reported to reduce both Aβ40 and Aβ42 and also decreased the thioflavin‐s positive Aβ aggregates through decreasing BACE1 and increasing ATP‐binding cassette transporter A1 (ABCA1) expression, which respectively decrease the production and increase the clearance of Aβ^78^ and also inhibited Aβ‐induced synaptic deficits^214^, ^215^ and prevented in vitro and in vivo stress‐induced hyperphosphorylation of τ.^216^ Unfortunately, this calpain inhibitor also has limited selectivity against other cysteine protease cathepsins B, K, L, S, and C thus preventing further investigation on this compound.^217^ Moreover, inhibition of cathepsins C, L, and S might induce serious side effects like immunosuppression while inhibition of cathepsins B and K is not detrimental.^218^, ^219^, ^220^, ^221^ This idea led to the very recent discovery of new calpain inhibitors derived from A‐705053 which retain the calpain inhibitory effect and with more selectivity. These compounds, A‐933548 and A‐953227, more specially the later, have more preclinical safety with in vivo anti‐AD profiles.^217^ But still further experiments are needed before considering the use of this calpain inhibitor as potential therapeutic treatment. Other recent developments result from another study that showed that inhibiting calpain‐2 by its selective inhibitor C2‐I enhanced learning and memory in both normal and genetically memory impaired mice by prolonging ERK activation.^196^ Another report revealed the downregulatory effect of both τ hyperphosphorylation and Aβ peptide production of synthetic chalcone derivatives^222^ conferring them a neuroprotective effect via decreasing p‐MAPK and BACE1 protein levels. Two other calpain inhibitors NYC438 and NYC488, have also been developed and showed promising results and had proven efficacy, potency, and safety.^223^

Collectively this suggested the promising potential of inhibiting calpain in AD therapeutics.

## 6 FUTURE DIRECTIONS

The two main hallmarks of AD are well established to be Aβ aggregate into SP and τ hyperphosphorylation forming NFTs. These are accompanied by a decline in cognitive functions including impaired memory and behavioral deficits. With Aβ deposit as the trigger of the disease, many of the primary AD therapies have focus on reducing Aβ load in the affected brain areas. Many of these strategies including monoclonal antibodies treatment against Aβ, vaccination with Aβ, Aβ aggregation inhibitors, β‐secretase interference, nerve growth factors, just to mention these, have succeeded in reducing the Aβ burden but often failed to improve the cognitive deficit and the resulting neurodegeneration. Considering this and also considering the fact that NFTs correlate more with the degree of the disease, therapeutic strategies that target both Aβ and τ hyperphosphorylation would be more efficient in treating the disease. From the summarized data above, it is clear that calpain hyperactivation is a big player in the generation and/or exacerbating these two AD hallmarks. Thus, targeting calpain either through downregulation or inhibition could be a way out. As reviewed earlier inhibiting calpain through overexpression of its endogenous inhibitor CAST and through the use of different synthetic inhibitors have provided promising results in neurodegenerative animal and cell models for the treatment of this conditions.

Calpain gene deletion has been proven to be lethal^224^ however suppression of calpain‐1 expression using specific probes prevented cell injury in human hepatic cancer cell lines induced by oxidative stress.^225^ In AD cell model other calpain inhibitors leupeptin, BDA‐410 and E64, have shown protective effects against cell death,^78^, ^162^, ^211^, ^212^ while MDL28170 had protective effect against excitotoxicity.^191^, ^210^ It is unfortunate to note that most of the currently used calpain inhibitors in AD therapeutics are limited due to their physical and/or chemical properties resulting in inefficient cellular penetration, selectivity, and kinetics. However, hope in this quest occurs from recent development of newly synthetic inhibitors, like A‐705053, A‐933548, A‐953227, NYC438, NYC488, and chalcone derivatives which have proven efficacy.^78^, ^188^, ^213^, ^214^, ^217^, ^222^, ^223^ Moreover, preliminary studies using these inhibitors in mouse and rat models of AD provided interesting results recovering cognitive functions when treatment was administered at an early age in these animals.^18^, ^212^, ^214^

Taken together, the data reviewed in this literature highlighted the undeniable involvement of calpain upregulation in AD pathology and the potential hope that reside in the development of calpain inhibitors as AD therapeutic tool and related neurodegenerative diseases. But there is still a long way to go in establishing the specificity, efficacy, safety, availability, and adequacy in the inhibitory effect of these compounds for the use in clinical trials and for the subsequent consideration for long‐term AD treatment, since calpains also play a normal physiological role as mentioned above. Supporting this is also a study where calpain inhibition led to the impairment of insulin release.^226^ It should also be noted that recent developments have provided evidence on the differential involvement of calpains in learning and memory and neurodegeneration where calpain‐1 has been reported to enhance learning and memory and neuroprotective while opposite effects have been attributed to calpain‐2.^196^, ^201^ Thus, specific targeting of these isoforms should be considered under different pathological conditions.

Clear and palpable evidence are provided from innumerable studies ranging from cell models to AD patients passing through animal models on the uncontestable role that upregulation of calpain plays in the pathogenesis of numerous neurodegenerative diseases among AD. This triggered a quest for the development of strategies aiming in decreasing calpain activity as treatment of AD and other neuronal injuries involving calpain hyperactivation. The result of this is the synthesis of numerous calpain inhibitors. However, many of these inhibitors are limited because of their insufficient cellular penetration, selectivity, and kinetics. Non‐the‐less, the idea that calpain inhibition could be a potential therapeutic strategy in neurodegenerative diseases still remain one of the promising option as supported by evidence from in vitro, in culture, and animal model studies using calpain inhibitors like MDL28170, A‐705053, A‐953227, NYC438, and NYC488 among others. However, it should be noted that these experiments were conducted in controlled environments with less or minimal influence of age‐related chronic diseases and in less complex organisms than humans.

## CONFLICTS OF INTEREST

The authors declare that there are no conflicts of interest.
